# Genome-Wide Analysis of the *LdERF* Gene Subfamily and Functional Characterization of *LdERF179* During Bulblet Regeneration in *Lilium*

**DOI:** 10.3390/plants15213265

**Published:** 2026-10-23

**Authors:** Xiaoping Xu, Shuxin Gong, Ying Tang, Yiqing Wang, Wenjie Guo, Shaozhong Fang, Muhammad Moaaz Ali, Faxing Chen, Chenglong Yang

**Affiliations:** 1 Fujian Provincial Key Laboratory of Agricultural Genetic Engineering, Biotechnology Research Institute, Fujian Academy of Agricultural Science, Fuzhou 350003, China; byxxp310107@163.com (X.X.); g2035878113@163.com (S.G.); yingtang0408@163.com (Y.T.); 18305921379@163.com (Y.W.); gwj73@126.com (W.G.); fangshaozhong1969@163.com (S.F.); 2 College of Horticulture, Fujian Agriculture and Forestry University, Fuzhou 350002, China; 3 Beijing Key Laboratory of Development and Quality Control of Ornamental Crops, Department of Ornamental Horticulture, China Agricultural University, Beijing 100193, China; 4 School of Tropical Agriculture and Forestry, Hainan University, Haikou 570228, China; muhammadmoaazali@yahoo.com

**Keywords:** *Lilium davidii* var. *unicolor*, AP2/ERF transcription factor, *LdERF179*, bulblet regeneration, auxin signaling, functional characterization

## Abstract

The AP2/ERF transcription factor family plays crucial roles in plant growth, development, and stress responses, as well as in organ regeneration. Here, a comprehensive genome-wide analysis of *ERF* subfamily of *Lilium davidii* var. *unicolor* (Lanzhou lily) was performed, and the functional role of a candidate gene, *LdERF179*, in bulblet regeneration was further investigated. A total of 206 *LdERF* genes were identified and phylogenetic evolution, chromosomal distribution, gene structure, conserved motifs, *cis*-regulatory elements, and collinearity were analyzed. Expression patterns suggested that there are 6 expression patterns of *LdERF* genes exhibited tissue-specific and stage-specific expression profiling during bulblet regeneration. *LdERF179* was highly expressed at the regeneration initiation (RI) stage (S1), suggested its potential involvement in the early morphogenesis of bulb regeneration. Subcellular localization analysis demonstrated that LdERF179 protein localized in the nucleus. The results of the virus-induced gene silencing (VIGS) functional validation analysis show that suppression of *LdERF179* significantly reduced bulblet formation efficiency from 40.67% to 9.43%. Further expression analysis indicated some key genes involved in auxin biosynthesis, transport, and signaling, and some regeneration-related genes showed decreased expression, such as TRV2-*LdERF179*, while increased expression was found in *LdERF17*9-OE (*LdASA1*, *LdAUX1*, *LdWOX11*). Therefore, we propose that *LdERF179* influenced the morphogenesis of bulblet regeneration by regulating the expression of auxin metabolism-related genes and key regeneration genes. However, further functional validation was required to clarify how *LdERF179* regulates auxin metabolism genes and key regeneration genes. This study provides new insights into the molecular regulatory network of lily bulblet regeneration and offers potential candidate genes for improving vegetative propagation efficiency in lilies.

## 1. Introduction

Plant growth and development require the continuous perception of environmental cues and the coordinated regulation of endogenous developmental programs to ensure successful growth, adaptation, and reproduction [1]. Transcription factors (TFs) are central regulators of gene expression that recognize and bind specific *cis*-regulatory elements in the promoters of target genes, thereby modulating downstream transcriptional networks [2]. Among plant TF families, the APETALA2/Ethylene-Responsive Factor (AP2/ERF) superfamily represents one of the largest and most functionally diverse groups and is widely distributed throughout the plant kingdom. Members of this superfamily are characterized by a highly conserved AP2 DNA-binding domain of approximately 60–70 amino acids, which typically consists of a three-stranded β-sheet followed by an α-helix [3]. According to the current classification system, the AP2/ERF superfamily is divided into five subfamilies: AP2, ERF, DREB, RAV, and Soloist [4]. Based on sequence characteristics of the AP2 domain and phylogenetic relationships, the ERF and DREB subfamilies are further divided into six groups each, designated B1–B6 and A1–A6, respectively [5]. This classification provides an important framework for understanding the evolutionary relationships and functional diversification of AP2/ERF family members.

The Ethylene-Responsive Factor (ERF) subfamily is one of the largest groups within the AP2/ERF superfamily and plays diverse roles in plant growth, development, and environmental adaptation. ERF proteins generally recognize specific *cis*-regulatory elements, including the GCC-box and dehydration-responsive element/C-repeat (DRE/CRT). DREB proteins mainly bind the DRE/C-repeat element (A/GCCGAC) and function in cold- and osmotic-stress signaling in the promoters of downstream target genes in the promoters of target genes [6]. Through their sequence-specific DNA-binding activity, ERF transcription factors regulate extensive transcriptional networks involved in morphogenesis, phytohormone signaling, and responses to biotic and abiotic stresses [7]. Recent studies have further highlighted the complexity of transcriptional regulatory networks underlying plant development, which may involve shared and gene-specific regulatory relationships, as well as auto- and cross-regulatory interactions among transcription factors [8]. Plant developmental transitions also involve dynamic crosstalk among multiple phytohormone signaling pathways rather than the independent action of individual hormones. Temporal transcriptomic analyses have revealed stage-dependent changes in auxin, cytokinin, gibberellin, abscisic acid (ABA), ethylene, jasmonate, and salicylate signaling, highlighting the coordinated and dynamic nature of hormonal regulation during development [9].

Numerous studies have demonstrated the diverse biological functions of ERF transcription factors in plant growth and development. For example, the Arabidopsis ERF transcription factor *TINY* regulates brassinosteroid-mediated plant development and drought responses [10]. These findings highlight the central roles of *ERF* in coordinating developmental processes with phytohormone signaling and environmental responses, suggesting that they may also function as important regulators of vegetative organ regeneration [11].

Increasing evidence has further implicated ERF family members in plant regeneration. In Arabidopsis, the expression of *ERF115* is rapidly upregulated upon tissue wounding, which promotes the cell cycle re-entry of differentiated cells, thereby contributing to stem cell proliferation and tissue regeneration [12]. Similarly, *WIND1* promotes cellular dedifferentiation and callus formation, facilitating subsequent organ regeneration [13]. In addition, *ERF109* regulates lateral root development by modulating auxin biosynthesis-related gene expression [14]. Collectively, these results suggest that ERF serves not only as key components of ethylene-responsive regulatory networks but also as important molecular regulators that integrate developmental and phytohormone signaling during organ regeneration [15].

Despite these advances, studies investigating the roles of ERF transcription factors in organ regeneration have focused predominantly on model species, particularly *Arabidopsis thaliana*, whereas their functions in bulbous plants remain poorly understood [16]. Several members of the AP2/ERF family have been identified and functionally characterized in *Lilium* species. For example, overexpression of *LoERF4* from Oriental lily (lily cultivar ‘Siberia’) in *Arabidopsis thaliana* significantly promoted root development [17]. In pink-flowered Oriental lilies, transient overexpression of *LhERF109* boosted anthocyanin accumulation and upregulated anthocyanin biosynthesis-related genes such as *LhMYB12*, suggesting that *LhERF109* positively regulates anthocyanin biosynthesis under high-temperature stress [18]. Conversely, in the Asiatic lily cultivar ‘Tiny Padhye’, *LhERF061* suppressed anthocyanin biosynthesis by repressing the expression of *LhMYBSPLATTER* and *LhDFR* through direct interaction with *LhMYBSPLATTER* [19]. These investigations reveal the functional versatility of AP2/ERF family members in *Lilium*, emphasizing their critical involvement in root development, anthocyanin biosynthesis, and environmental stress responses [18,19,20]. Nevertheless, the roles of ERF in lily bulblet regeneration are still poorly understood. In particular, the *LdERF* members associated with different stages of bulblet regeneration and their potential regulatory roles remain largely unknown.

Lanzhou lily (*Lilium davidii* var. *unicolor*), a perennial monocotyledonous herb of the genus *Lilium* in the family Liliaceae, holds significant edible, medicinal, and ornamental value. It is characterized by large bulbs with thick, white fleshy scales and a distinctive sweet flavor, earning it the name “sweet lily” [21]. Recognized as a National Geographical Indication Product of China, Lanzhou lily is a characteristic agricultural product of Gansu Province. Its bulb serves not only as the primary edible and medicinal organ but also as the principal structure for vegetative propagation [22].

However, Lanzhou lily exhibits a low natural propagation coefficient and requires a prolonged cultivation period to produce mature bulbs [23,24]. Although scale cutting remains the predominant propagation method in commercial production, this approach is characterized by a long production cycle and is highly influenced by environmental conditions. Tissue culture and in vitro bulblet regeneration therefore provide promising alternatives for the rapid propagation and multiplication of elite germplasm resources, with bulblet regeneration efficiency representing a key determinant of propagation success [25]. Accordingly, elucidating the molecular mechanisms underlying bulblet regeneration and identifying key regulatory genes involved in this process are essential for improving propagation efficiency, establishing efficient regeneration and genetic transformation systems, and accelerating the genetic improvement of lily cultivars [26].

In this study, we conducted comprehensive genome-wide identification and systematic characterization of the *ERF* subfamily in Lanzhou lily using its genome sequence. Comprehensive analyses, including phylogenetic relationships, chromosomal distribution, conserved synteny, gene architecture, conserved motifs, and *cis*-regulatory elements, were conducted to characterize the *LdERF* gene family. Among these genes, *LdERF179* was selected for further functional characterization because of its relatively high expression throughout bulblet regeneration, particularly during the early stages, suggesting a potential association with bulblet formation. In addition, *LdERF179* exhibited high quantitative sequence-identity value with *AtERF1* from *Arabidopsis thaliana* and its nuclear localization further supported its potential role as a transcriptional regulator. Therefore, *LdERF179* was chosen for subsequent functional analysis. Its role in bulblet regeneration was subsequently investigated through virus-induced gene silencing (VIGS), together with expression analyses of regeneration-related and phytohormone-associated genes. This study provides insights into the potential role of *LdERF179* in bulblet regeneration and its possible contribution to improving regeneration efficiency in lily.

## 2. Results

### 2.1. Genome-Wide Identification of the ERF Subfamily from Lanzhou Lily

A total of 206 ERF subfamily genes were identified from the *Lilium davidii* var. *unicolor* genome through HMM-based searches, BLASTP analysis, and conserved domain verification. Both HMM and BLASTP searches used an E-value threshold of 1 × 10^−10^, and redundant protein sequences were removed using CD-HIT at a sequence identity threshold of 90%. After removing redundant sequences, the identified genes were designated *LdERF1*–*LdERF206* based on their chromosomal locations (Table S1, Figure S1). Each identified gene possessed a complete AP2 DNA-binding domain and an intact ORF, lacking any truncated sequences or missing conserved domains, which confirmed their suitability for further analyses.

Physicochemical characterization of the predicted LdERF proteins revealed substantial variation among family members (Table S1). The protein lengths varied from 95 amino acids in *LdERF78* to 543 amino acids in *LdERF79*, with molecular weights between 10.51 and 60.89 kDa. Predicted pI values ranged from 4.37 (*LdERF79*) to 11.49 (*LdERF*118), averaging 7.56. Among the 206 proteins, 112 (54.4%) had pI values ≥ 7, whereas 94 (45.6%) had pI values < 7, indicating that both basic and acidic proteins are represented within the family.

GRAVY values varied from −1.201 to −0.035, averaging −0.522. The consistently negative GRAVY scores across all LdERF proteins indicate their hydrophilic nature. The aliphatic index, a predictor of thermostability, ranged from 41.94 for *LdERF78* to 92.26, averaging 67.04, which reflects moderate variation in the predicted thermostability of these proteins. The instability index varied between 21.18 and 88.15, averaging 55.77, suggesting that most LdERF proteins were predicted to be unstable.

WoLF PSORT-based subcellular localization prediction suggested that *LdERF179* is likely nuclear.

### 2.2. Phylogenetic Evolution of the LdERF Subfamily

**Figure 1:**
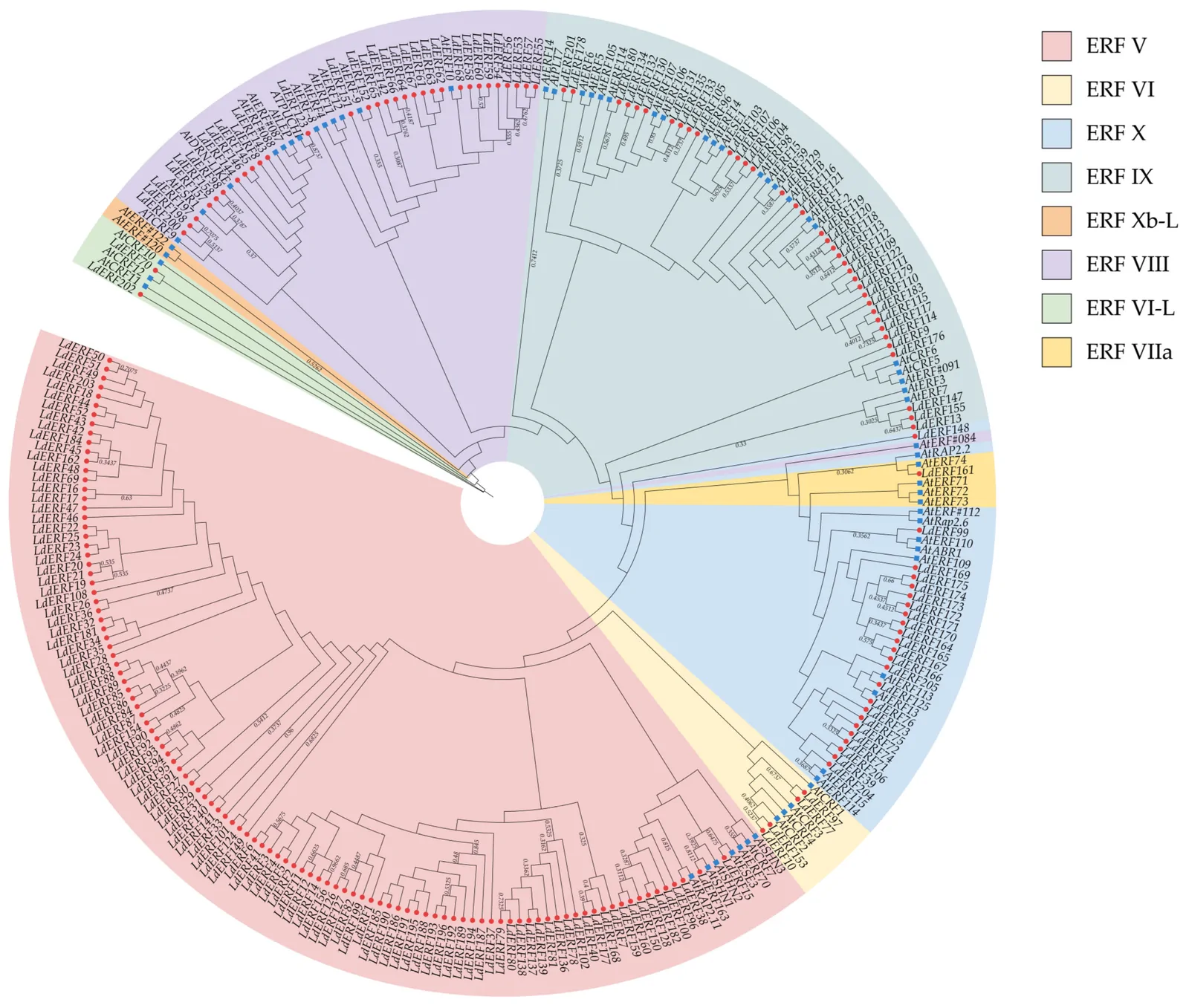
Phylogenetic relationships of ERF proteins from *Lilium davidii* var. *unicolor* and *Arabidopsis thaliana*. Different subgroups are indicated by distinct background colors.

To investigate the phylogenetic connections of the *LdERF* subfamily, we constructed a phylogenetic tree using ERF protein sequences from Lanzhou lily and *Arabidopsis thaliana*. Phylogenetic analysis of the 206 LdERF proteins together with ERF reference proteins from *Arabidopsis thaliana* was performed using the maximum likelihood (ML) method, with 1000 bootstrap replicates to evaluate the support for the inferred clades (Figure 1). According to the established classification system [27], the ERF proteins were divided into eight distinct clades: ERF V (106 genes), ERF VI (4 genes), ERF VIIa (1 gene), ERF VIII (31 genes), ERF IX (38 genes), ERF X (24 genes), and ERF VI-L (2 genes). For instance, the bootstrap support values for the nodes grouping *AtERF114* with *LdERF180*, *AtESE3* with *LdERF15*, and *AtRAP2.11* with *LdERF38* were 56%, 64.75%, and 81.5%, respectively.

Among these clades, ERF V contained the largest number of LdERF members and represented a markedly expanded lineage, whereas ERF Xb-L contained the fewest LdERF members. Several LdERF proteins clustered with their *Arabidopsis* homologs in well-supported monophyletic clades, indicating a relatively high degree of evolutionary conservation. Conversely, some lily-specific clusters failed to associate with *Arabidopsis* ERF proteins, implying lineage-specific gene expansion and potential functional diversification of the ERF gene family throughout lily evolution.

### 2.3. Chromosomal Distribution and Synteny Analysis

Chromosomal location mapping showed that the 206 *LdERF* genes were non-uniformly distributed across the 12 chromosomes of Lanzhou lily (Figure S1). A relatively high density of *LdERF* genes was observed on LG02, LG03, LG04, and LG11, where several genes were arranged in distinct clusters. Among these chromosomes, LG02 contained the largest number of *LdERF* genes, including a cluster comprising 28 genes. In contrast, relatively few *LdERF* genes were located on LG05, LG06, and LG12, with LG05 containing only five genes. These results demonstrate the uneven chromosomal distribution of the *LdERF* gene family, with certain chromosomes and regions containing relatively high densities of family members.

**Figure 2:**
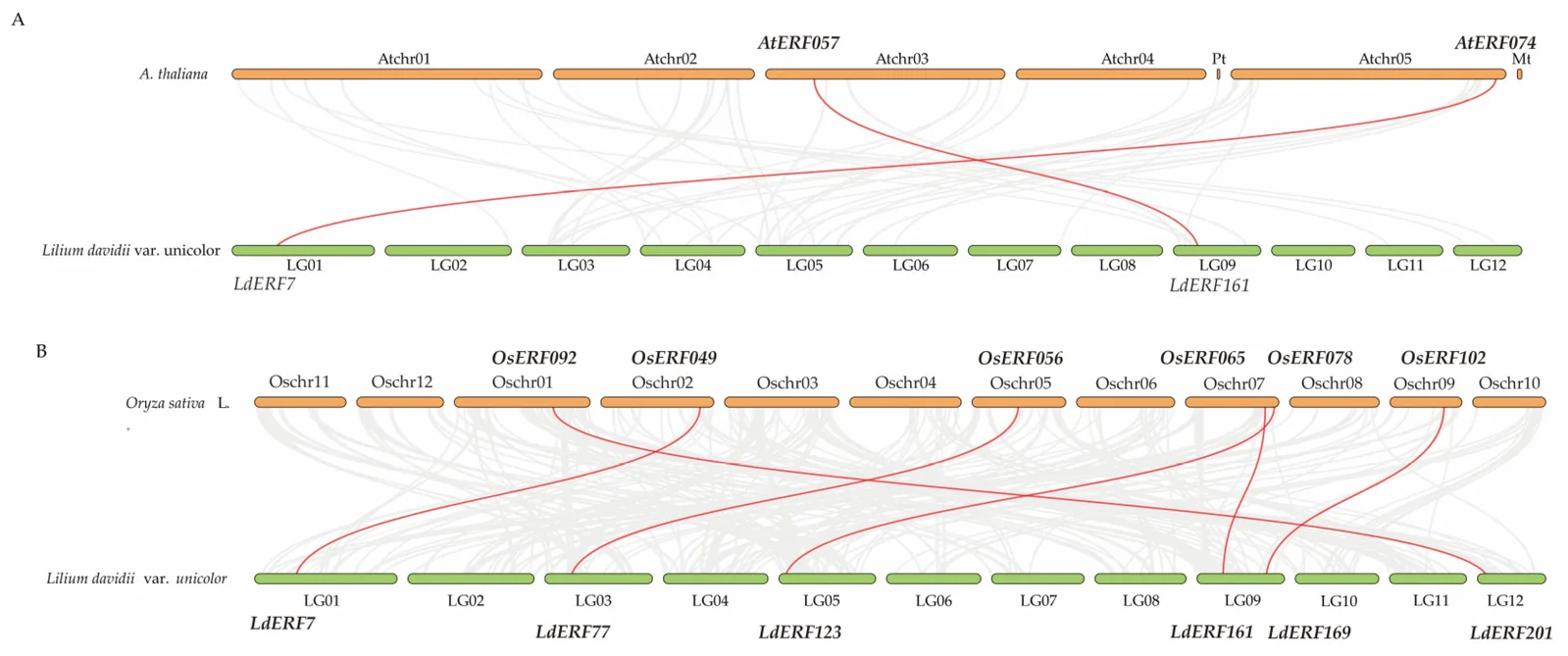
Synteny analysis of *LdERF* genes among *Lilium davidii* var. *unicolor*, *Arabidopsis thaliana* (**A**) and *Oryza sativa* (**B**). Red lines indicate conserved AP2/ERF gene pairs between *Lilium davidii* var. *unicolor* and the other species, whereas grey lines indicate syntenic genomic blocks. Labeled genes indicate the homologous gene pairs between *Lilium davidii* var. *Unicolor* and *Arabidopsis*/rice.

To explore the evolutionary connections of *LdERF* genes with those in other plants, synteny analyses were conducted between Lanzhou lily and both *A. thaliana* and *Oryza sativa* (Figure 2). Two pairs of orthologous genes were identified between Lanzhou lily and *A. thaliana*, whereas six pairs of orthologous genes were detected between Lanzhou lily and *O. sativa* (Table S2). These conserved collinear associations suggest that a subset of *LdERF* genes has been retained during the evolution of different plant lineages.

### 2.4. Analysis of Gene Structure, Conserved Domains, and Conserved Motifs of the LdERF Gene Family

Gene structure analysis revealed that *LdERF* genes generally exhibited relatively simple exon–intron organizations (Figure S2). Most genes contained one to five exons, although considerable variation in gene length was observed, with 22 *LdERF* members containing exceptionally long introns. Among them, 145 *LdERF* genes contained a single exon, accounting for more than half of the family, such as *LdERF1*, *LdERF123* and *LdERF179*; 42 *LdERF* genes contained two exons, such as *LdERF29*, *LdERF40* and *LdERF59*; 14 contained three exons; 4 contained five exons, such as *LdERF51*, *LdERF167* and *LdERF175*; and only one gene (*LdERF176)* contained four exons.

Conserved domain analysis indicated that all LdERF proteins contain the characteristic AP2 DNA-binding domain, confirming the high conservation of the defining DNA-binding region within this gene family (Figure S3).

**Figure 3:**
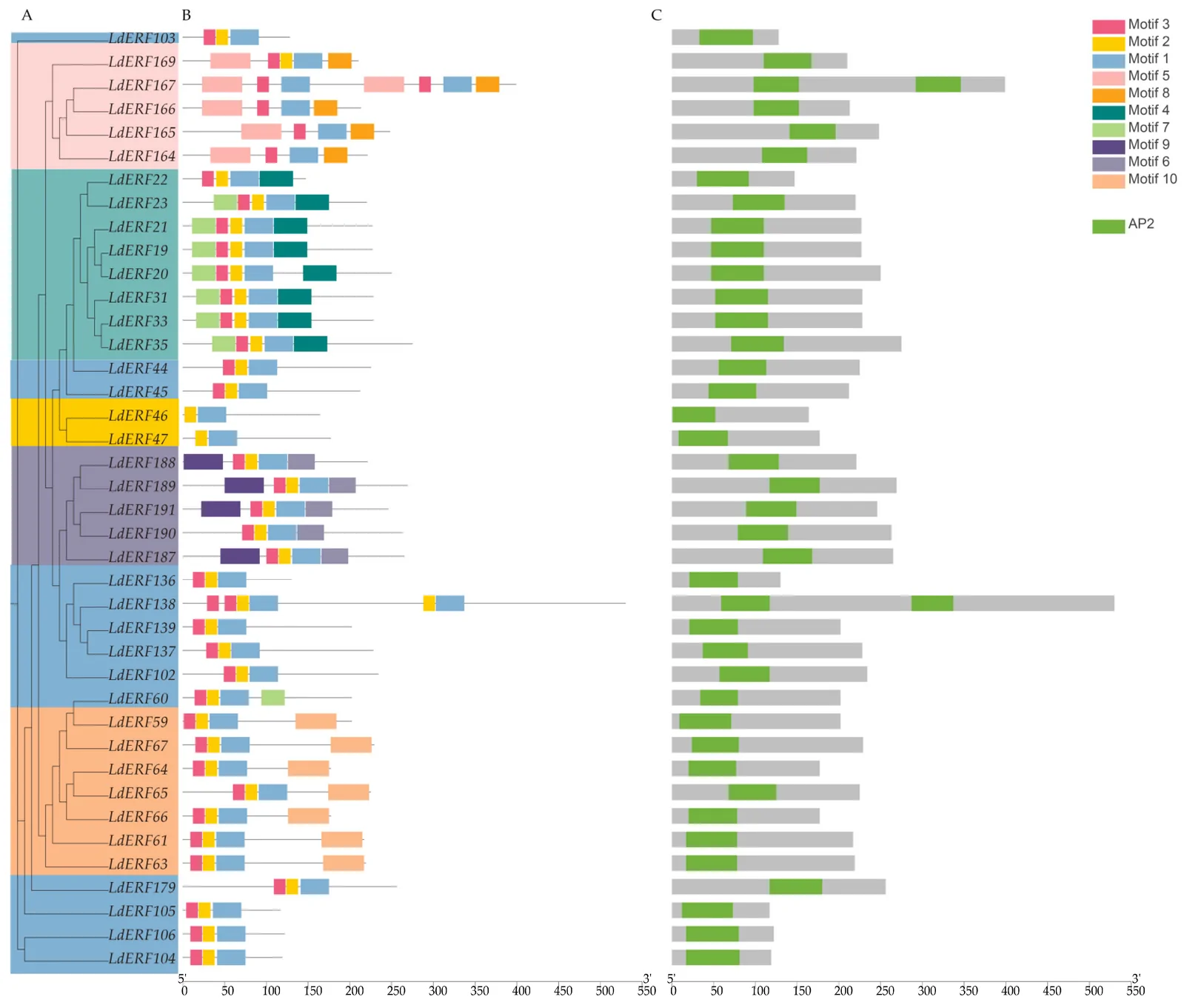
Conserved motif composition and domain organization of some major LdERF proteins in *Lilium davidii* var. *unicolor*. (**A**) Phylogenetic tree of the LdERF subfamily. (**B**) Distribution of conservative motifs in the LdERF subfamily. (**C**) Distribution of conservative domains in the LdERF subfamily.

A total of 10 conserved motifs were identified in the LdERF proteins. Motifs 1 and 2 were present in most family members and corresponded to conserved regions of the AP2 domain, representing characteristic motifs of the LdERF family (Figure 3A–C).

### 2.5. Cis-Regulatory Element Analysis

*Cis*-regulatory element analysis of 206 *LdERF* gene promoters uncovered a wide variety of elements linked to plant growth and development, phytohormone signaling, light perception, and abiotic stress responses (Figure S4, Table S3).

**Figure 4:**
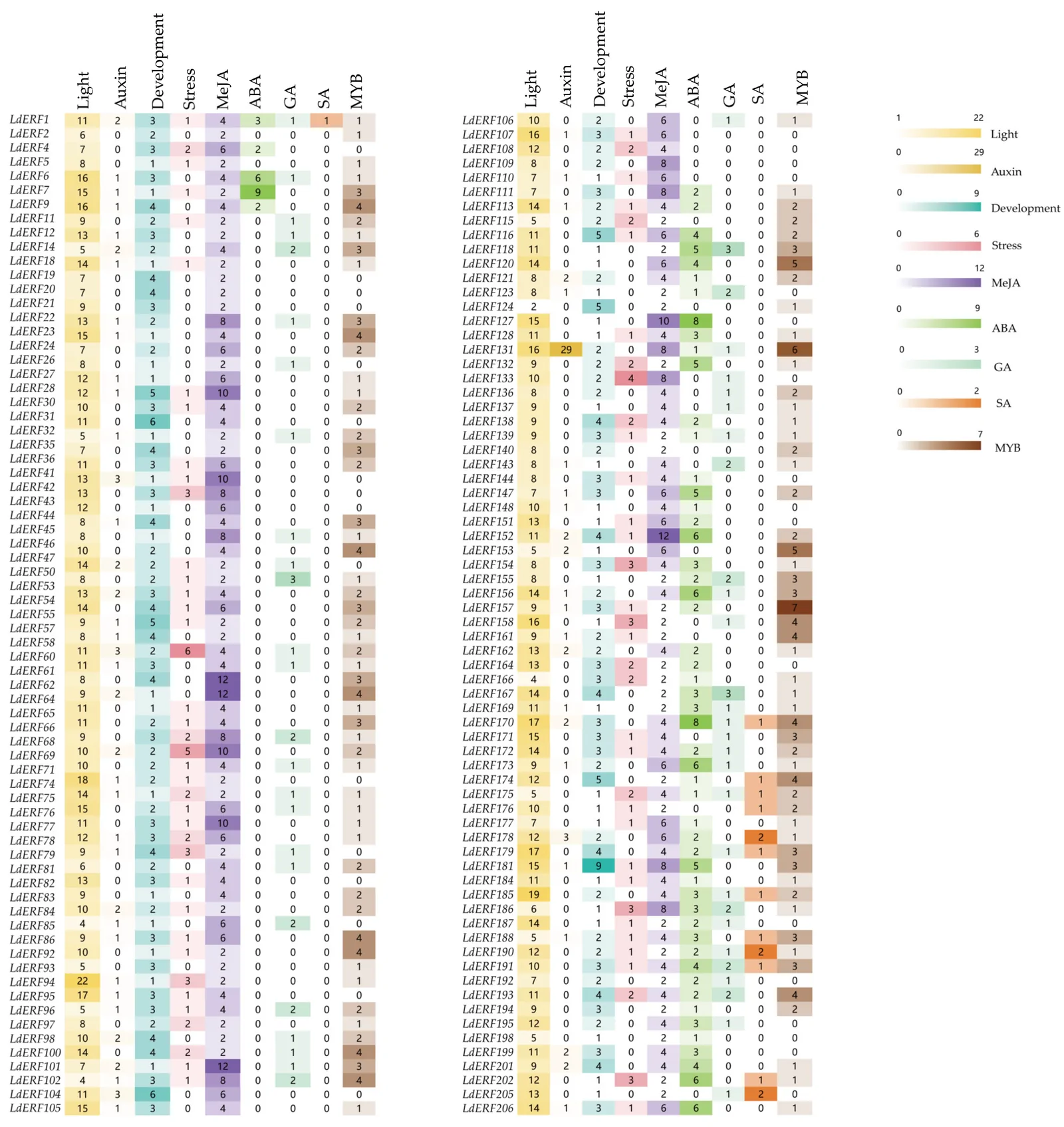
The number of predicted *cis*-acting elements distributed on promoter regions of *LdERF* genes. Values indicate the number of independent predicted sites for each response type in each gene; records with the same gene, response type, position, and strand were counted once. Color intensity represents site abundance, with separate color scales for the nine functional categories.

To further identify which *LdERF* members may be involved in growth and development and in trauma-induced regeneration of bulblets, 141 LdERF containing *cis*-regulatory elements for the growth and development and Me-JA response were selected for promoter *cis*-regulatory element analysis. Generally, light-responsive elements were the most abundant, followed by elements associated with abiotic stress and phytohormone responses. Stress-related elements included those linked to low-temperature, drought, and salt responses, whereas hormone-responsive elements were associated with ABA, JA, and GA signaling (Figure 4). For instance, *LdERF131* was characterized predominantly by auxin-responsive elements (29), whereas *LdERF61* and *LdERF62* were characterized predominantly by MeJA-responsive elements (12); *LdERF179* and *LdERF181* contained both MeJA- and development-related elements. When integrated with the phylogenetic analysis, members within the same ERF subgroup generally exhibited similar *cis*-element compositions, particularly with respect to light-, hormone-, and stress-responsive elements. In contrast, substantial differences in *cis*-element composition were observed among different subgroups, suggesting potential regulatory and functional divergence of *LdERF* genes during evolution.

### 2.6. Tissue-Specific and Bulblet Regeneration-Associated Expression Patterns of the LdERF Subfamily

**Figure 5:**
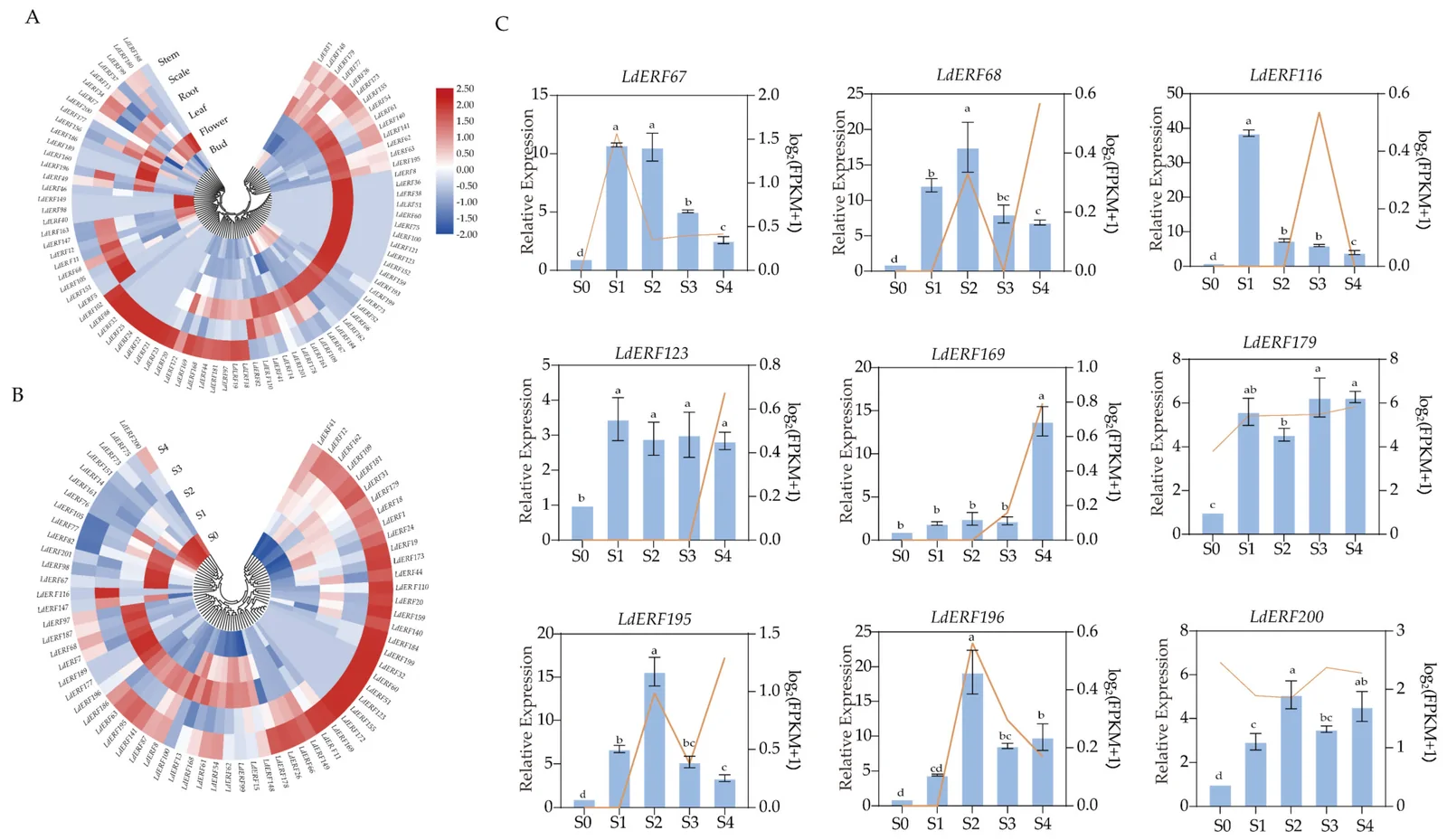
Expression profiles of the *LdERF* subfamily in different tissues and during bulblet regeneration. (**A**) Expression heatmap of *LdERF* homologs during bulblet regeneration in lily. (**B**) Expression heatmap of *LdERF* homologs in different tissues of lily. (**C**) RT—qPCR verification of expression trends for specific *LdERF* genes during bulblet regeneration of Lanzhou Lily. The line chart shows the standardized FPKM values (log_2_(FPKM+1)). Letters mark significant differences by one-way ANOVA (*p* < 0.05).

Transcriptome-based expression analysis of *LdERF* homologs using the dataset from the lily cultivar ‘Siberia’, which was referenced from the *L. davidii* var. *unicolor* genome for gene annotation, revealed diverse expression patterns in different tissues and the bulblet regeneration stages (Figure 5A,B). After excluding genes with undetectable expression, 85 *LdERF* genes were extracted in different tissues, whereas 68 genes exhibited differential expression during bulblet regeneration. There were 6 expression profiles across different tissues (Figure 5A). Among them, most members of *LdERF* (*LdERF179*, *LdERF1 LdERF148* and *LdERF77*) were highly expressed in nutritive organs, such as root (*LdERF25*, *LdERF70*, and *LdERF169)* and stem (*LdERF179*, *LdERF88*, and *LdERF25*), followed by scales (*LdERF1*, *LdERF82*, and *LdERF151*) and leaf (*LdERF160*, *LdERF186*, and *LdERF189*), and finally bud (*LdERF46*, *LdERF149*, and *LdERF98*). Meanwhile, 5 *LdERF* members showed higher expression in flower (such as *LdERF99*, *LdERF168*, and *LdERF180*). These results suggest that different *LdERF* genes may perform distinct tissue-specific functions during plant growth and development.

Furthermore, 68 *LdERF* genes exhibited distinct expression dynamics from S0 to S4. There were 6 expression profiles during different bulblet regeneration stages. Firstly, 32 LdERF members showed upregulation during bulblet regeneration, and high expression was found in S3 and S4, such as in *LdERF41*, *LdERF12*, and *LdERF162*. Next, there were 15 *LdERF* members highly expressed in the promeristem formation (PF) stage (S2), such as *LdERF179*, *LdERF195*, and *LdERF141*. Thirdly, 8 *LdERF* members were highly expressed in the regeneration initiation (RI) stage (S1), such as *LdERF98*, *LdERF67*, and *LdERF201*. Finally, a few *LdERF* members (6) showed increased expression in the scale induction (SI) stage (S0) and a gradual decline during regeneration, such as *LdERF7*, *LdERF177*, and *LdERF97*. Other genes displayed fluctuating expression patterns, including transient upregulation followed by downregulation or the opposite trend. A small number of *LdERF* genes (*LdERF62* and *LdERF61*) maintained consistently low expression or showed limited variation throughout the regeneration process. These results indicate that *LdERF* homologs exhibit diverse transcriptional dynamics during bulblet regeneration.

To further characterize the expression patterns of *LdERF* genes during bulblet regeneration in *Lilium davidii* var. *unicolor*, the expression levels of nine selected genes were examined by RT-qPCR. Among the validated genes, *LdERF67* and *LdERF95* reached peak expression during S1–S2, *LdERF169* was markedly upregulated at S4, and *LdERF116* and *LdERF196* exhibited stage-specific expression patterns during regeneration (Figure 5C). Notably, *LdERF179* maintained relatively high expression during the early stages of bulblet regeneration (S0–S2) (Table S4), whereas most other *LdERF* genes exhibited relatively low or only modestly fluctuating expression during the same period. Moreover, *LdERF179* showed relatively high expression in the basal region of bulb scales and floral tissues but comparatively low expression in leaves and stems, indicating a distinct tissue-specific expression pattern. Together with its relatively high expression during the early stages of bulblet regeneration and its distinct tissue-specific expression pattern, these results led to the selection of *LdERF179* for subsequent functional characterization.

### 2.7. LdERF179 Subcellular Localization

To experimentally ascertain the subcellular localization of *LdERF179*, the recombinant construct 35S::*LdERF179*-eGFP and the control construct 35S::eGFP were transiently expressed in onion epidermal cells.

**Figure 6:**
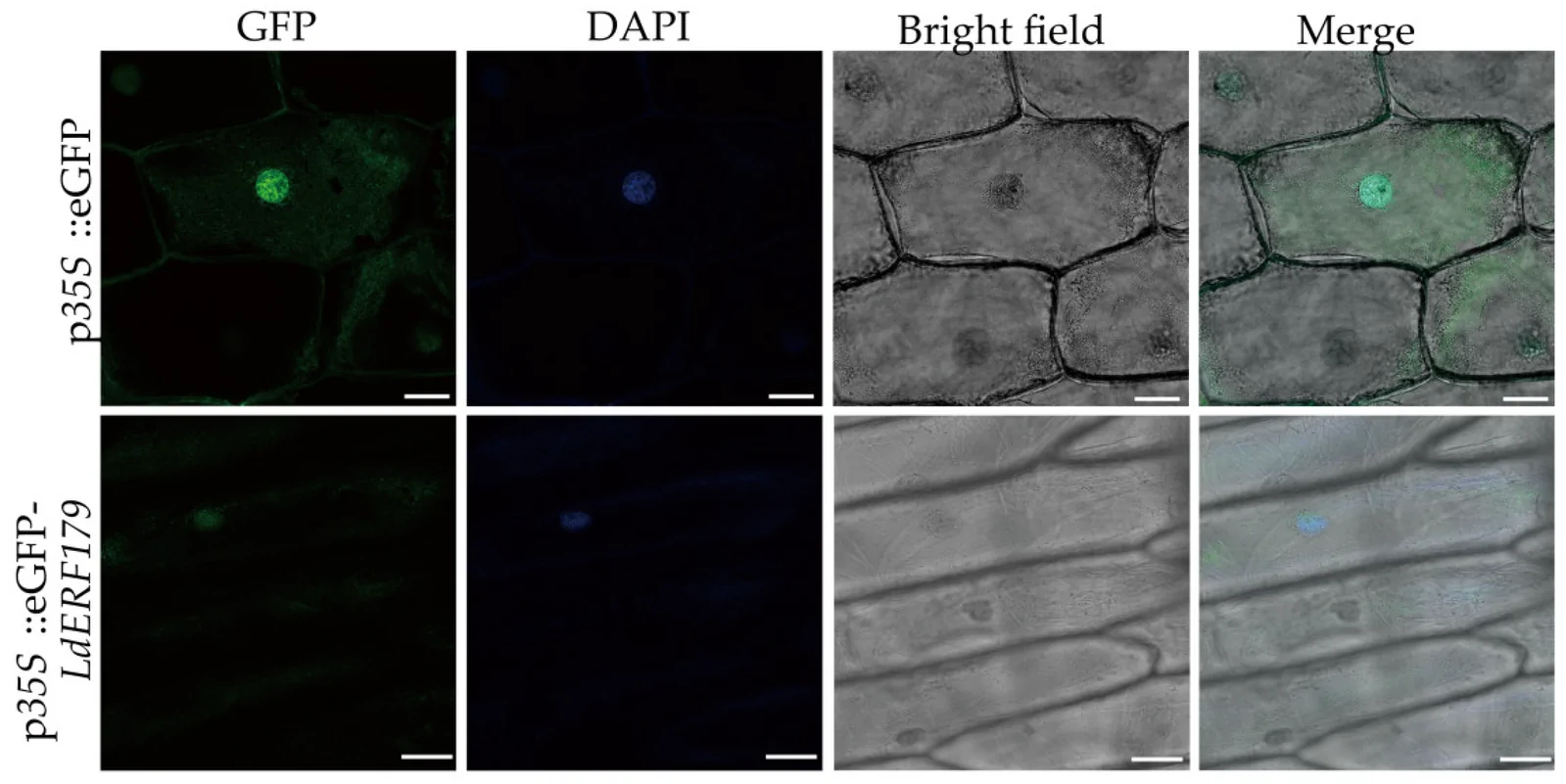
Subcellular distribution of *LdERF179* in onion epidermal cells. The *LdERF179*-eGFP nuclear localization was observed for the fusion protein of inner epidermal cells of onion. DAPI served as a nuclear marker. The blue fluorescence indicates nuclear localization staining with DAPI. Scale bar: 50 μm.

In the control group, eGFP fluorescence was distributed throughout both the cytoplasm and the nucleus, and overlapped with the DAPI nuclear staining signal. Conversely, the fluorescence of *LdERF179*-eGFP fusion protein exhibited completely co-localized in the nucleus, perfectly overlapping with the DAPI signal, while no significant fluorescence was detected in the cytoplasm (Figure 6). These findings confirm the nuclear localization of LdERF179, aligning with its predicted function as a TF.

### 2.8. Silencing of LdERF179 Inhibited Bulblet Regeneration in Lanzhou Lily

To explore the role of *LdERF179* in bulblet regeneration, the recombinant TRV2-*LdERF179* construct was introduced into Lanzhou lily scales using VIGS. Samples were collected 21 days after inoculation. PCR amplification with CP-specific primers confirmed successful viral infection of the TRV coat protein (CP) gene (Figure S5).

**Figure 7:**
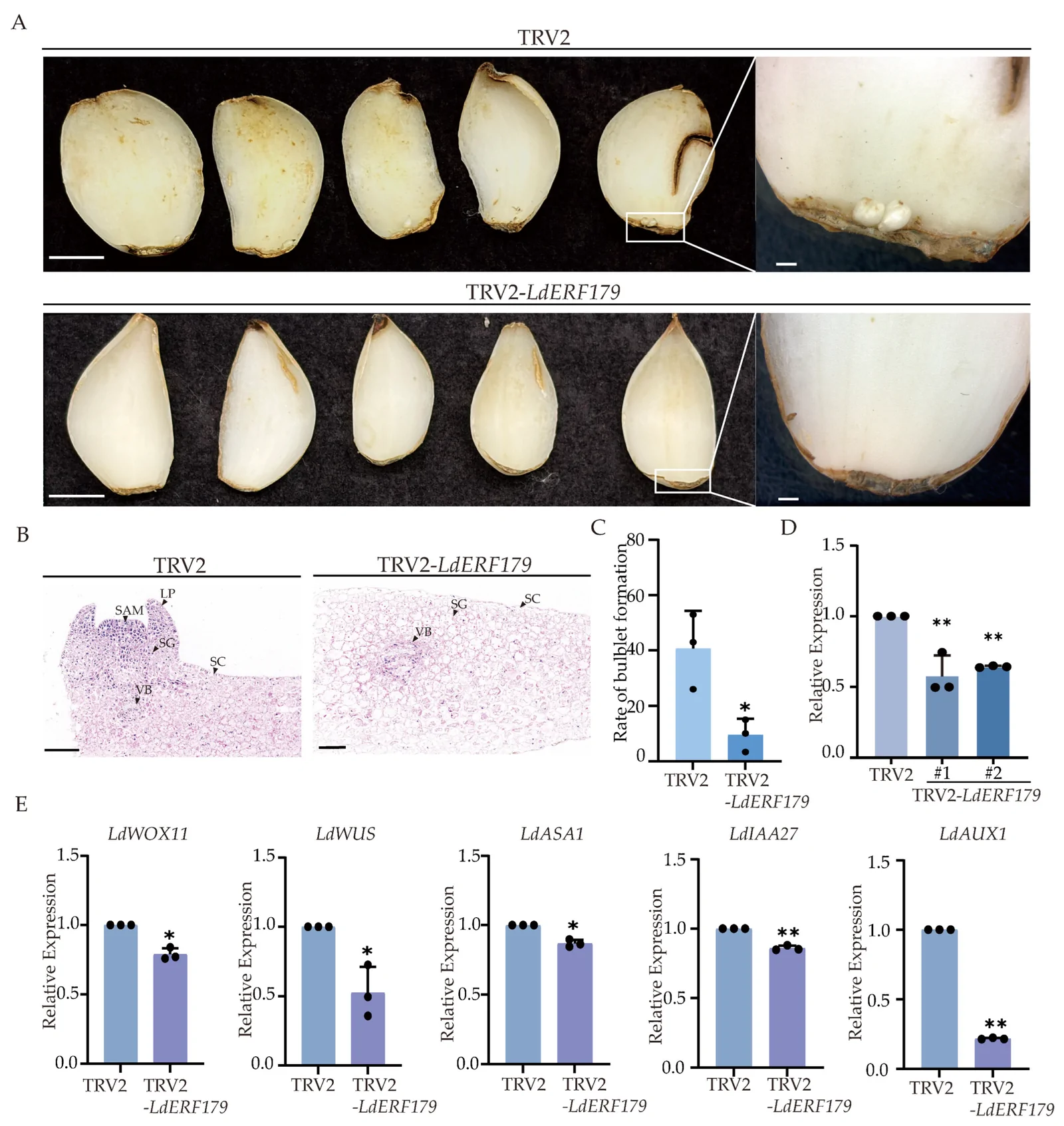
Silencing of *LdERF179* inhibited bulblet regeneration in Lanzhou Lily. (**A**) Phenotypes of lily scale bases after silencing *LdERF179* and the empty TRV2 control. Scale bar = 1 cm for overview panels and 1 mm for magnified detail panels. (**B**) Paraffin sections of lily scale bases infected with TRV2-*LdERF179* and empty TRV2 vector, respectively. Scale bar = 1 mm. (**C**) The bulblet regeneration frequency of bulblets in silenced plants and empty TRV2 control plants. (**D**) Relative expression level of *LdERF179* in silenced plants. (**E**) Relative expression levels of downstream target genes regulated by *LdERF179* in silenced plants. (Three independent biological replicates were performed, with 30 scales per construct per replicate after 21 d of culture, asterisks from Student’s *t*-test: * *p* < 0.05, ** *p* < 0.01).

Real-time quantitative PCR (RT-qPCR) analysis showed that *LdERF179* expression levels were markedly decreased in the TRV2-*LdERF179* treatment compared with the TRV2 empty-vector control, confirming effective silencing of the target gene (Figure 7D).

Phenotypic analysis further showed that bulblet regeneration was markedly inhibited following *LdERF179* silencing (Figure 7A). The bulblet regeneration frequency decreased from 40.67% in the TRV2 control to 9.43% in the TRV2-*LdERF179* treatment (Table S5, Figure 7C), supporting *LdERF179*-promoted bulblet regeneration in Lanzhou lily.

To further investigate the molecular basis underlying *LdERF179*-mediated bulblet regeneration, RT-qPCR was used to analyze the expression of genes involved in auxin biosynthesis, transport, signaling, and meristem development (Figure 7E). Transcript abundance levels of the auxin biosynthesis gene *LdASA1*, the auxin signaling gene *LdIAA27*, and the auxin influx carrier gene *LdAUX1* were significantly reduced following *LdERF179* silencing. In addition, the regeneration-associated genes *LdWOX11* and *LdPIN1* were also significantly downregulated compared with the control. These results indicate that *LdERF179* silencing is associated with altered expression of genes related to auxin homeostasis and developmental regulation during bulblet regeneration. However, the observed expression changes do not establish direct transcriptional regulation of these genes by *LdERF179*.

Histological observations further supported the inhibitory effect of *LdERF179* silencing on bulblet regeneration. Paraffin sections of the basal regions of infected scales revealed clear differences in tissue organization between the two treatments (Figure 7B). In the TRV2 control, compact meristematic protrusions composed of actively dividing cells were clearly observed at the scale base. In contrast, tissues in the TRV2-*LdERF179* treatment consisted of loosely arranged and enlarged cells, with only limited protrusion formation, indicating severe suppression of bulblet regeneration. Collectively, these observations support the involvement of *LdERF179* in bulblet regeneration and suggest that it may be associated with cellular proliferation and the initiation of regenerative structures.

### 2.9. Overexpression of LdERF179 Upregulates the Expression of Auxin Metabolism-Related Genes and Key Regeneration Genes in Lanzhou Lily

**Figure 8:**
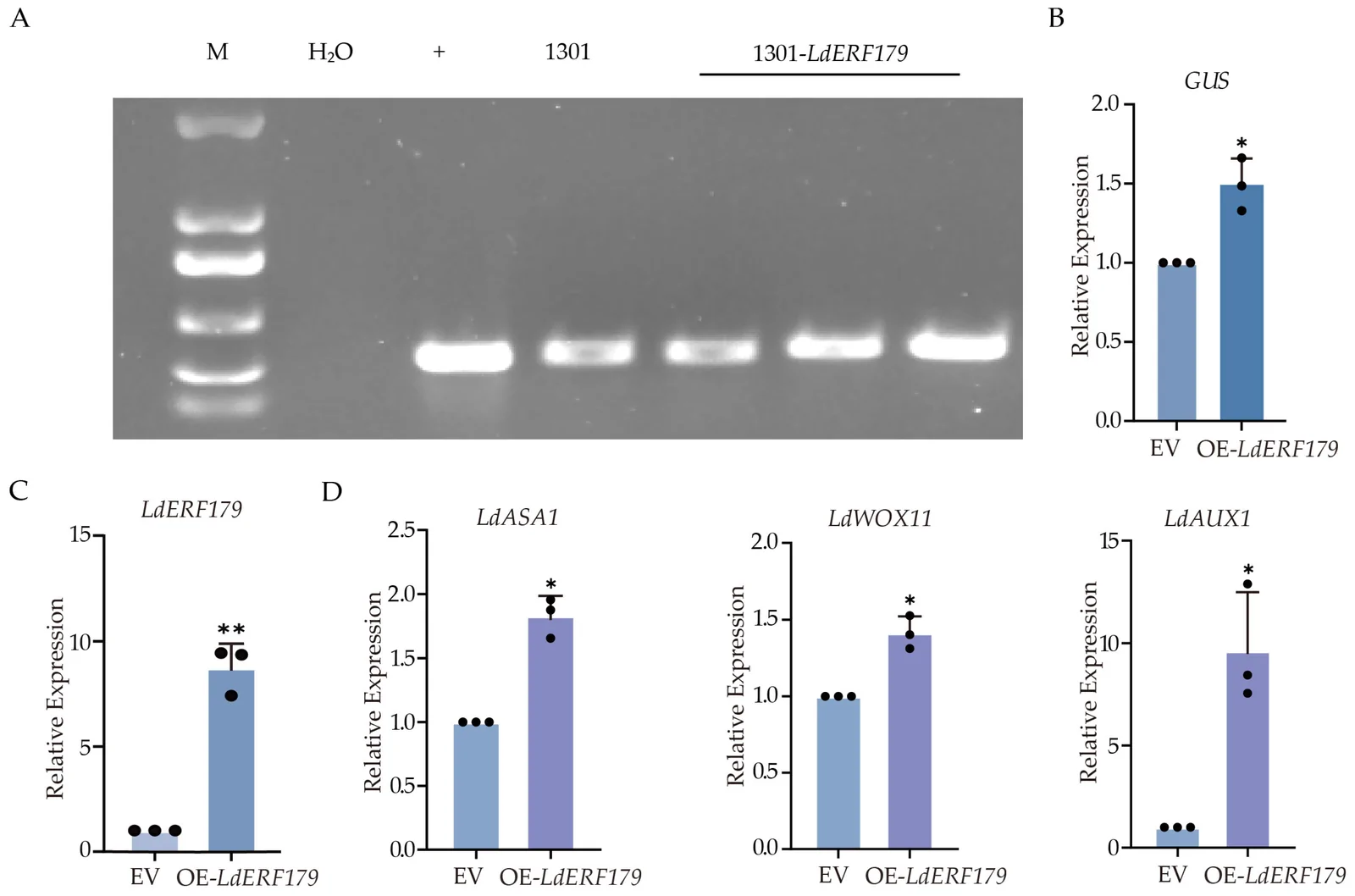
Overexpression of *LdERF179* upregulates the expression of auxin metabolism-related genes and key regeneration genes in Lanzhou Lily. (**A**) indicates PCR detection of the *GUS* gene. (**B**) GUS expression level in overexpressing plants. (**C**) *LdERF179* relative expression level in overexpressing plants. (**D**) Relative expression levels of auxin metabolism- and regeneration-related genes in *LdERF179* overexpressing plants. Asterisks indicate significant differences relative to the empty vector (EV) control (Student’s *t*-test: * *p* < 0.05, ** *p* < 0.01).

To further verify whether *LdERF179* regulated auxin metabolism and key regeneration genes during bulblet regeneration, a 35S::*LdERF179* overexpression construct was generated using the pCAMBIA1301 vector, while the empty pCAMBIA1301 vector served as the EV control. The construct was introduced into lily scales using the same Agrobacterium-mediated transformation procedure described above. PCR amplification of the vector-borne *Hyg* and *GUS* marker genes verified successful transformation (Figure S6; Figure 8A). RT-qPCR results indicated that *GUS* and *LdERF179* expression levels were significantly higher in OE-*LdERF179* tissues than in the EV control, confirming successful overexpression of *LdERF179* (Figure 8B,C).

To further explore the expression alterations linked to the *LdERF179* overexpression, RT-qPCR was employed to assess the transcript levels of specific genes. Compared with the EV control, overexpression of *LdERF179* significantly increased the expression of *LdASA1*, *LdWOX11*, and *LdAUX1* (Figure 8D). Among these genes, *LdAUX1* exhibited the greatest increase, reaching an expression level approximately 10-fold greater than that in the EV control. These findings indicated that *LdERF179* overexpression was associated with increased expression of genes related to auxin-associated processes and bulblet regeneration. However, because quantitative phenotypic data on bulblet regeneration were not obtained in the overexpression experiment, these expression changes alone are insufficient to conclude that *LdERF179* overexpression promotes bulblet regeneration. Furthermore, whether and how *LdERF179* directly regulates these genes remains to be determined.

## 3. Discussion

### 3.1. Functional Diversification of the LdERF Gene Family in the Lanzhou Lily Genome

By recognizing and binding to specific *cis*-regulatory elements in target gene promoters, TFs regulate gene expression, thereby playing crucial roles in plant growth, development, and stress responses. [18]. As one of the largest and most functionally diverse groups within the AP2/ERF superfamily, ERF proteins participate extensively in developmental regulation, phytohormone signaling, and adaptation to biotic and abiotic stresses. While AP2/ERF family members have been reported in many plant species, considerable variation exists in the size and composition of this gene family among different plant genomes.

In the present study, a total of 206 *LdERF* genes were detected in the *Lilium davidii* var. *unicolor* genome, which is substantially greater than the numbers documented in other plant species, including *A. thaliana*, rice, and grapevine. The enlargement of the *LdERF* family might be linked to the remarkably vast and intricate genome of Lanzhou lily. Prior research has indicated that the genome of Lanzhou lily is roughly 36.68 Gb in size and is characterized by abundant repetitive sequences and unusually long introns, genomic features that may have facilitated gene duplication and family expansion. Consistent with this possibility, the 12 chromosomes exhibited an uneven distribution of *LdERF* genes, with multiple genes forming distinct clusters.

Evolutionary relationship analysis further classified 206 LdERF proteins into eight subgroups: ERF V, ERF VI, ERF VI-L, ERF VIIa, ERF VIII, ERF IX, ERF X, and ERF Xb-L. Proteins in the same subgroup typically exhibited comparable conserved motif patterns and exon–intron structures, indicating their close phylogenetic affinity. In contrast, substantial differences in motif composition were observed among different subgroups, suggesting that functional divergence accompanied the evolutionary expansion of the *LdERF* family. Comparable trends have been documented in *Arabidopsis*, rice, and maize, indicating that although ERF proteins retain a conserved AP2 DNA-binding domain, diversification of other sequence regions may have contributed to the acquisition of distinct regulatory functions in different species. Within the LdERF family, Motifs 1 and 7 correspond to the conserved AP2 DNA-binding domain and are shared by the entire family. In contrast, Motifs 2–6 and Motifs 8–10 are located outside the AP2 domain, and their composition varies significantly among the seven subfamilies. Since the AP2 domain determines only DNA-binding specificity, while others conserve motifs out of the AP2 DNA-binding domain, which might serve other functions, such as transcriptional activation/repression, nuclear localization, and protein stability, these subgroup-specific extra-domain motifs may constitute the molecular basis for the functional differentiation of the LdERF family and may correspond to subgroup-specific expression patterns and hormone response characteristics.

Further promoter analysis indicated that *LdERF* genes harbor various *cis*-regulatory elements linked to light perception, phytohormone signaling, and abiotic stress responses, such as those responsive to ABA, MeJA, and auxin. The distribution of the presence of these regulatory elements implies that *LdERF* genes may respond to the signal transduction of these abiotic stresses. Together, the phylogenetic, structural, and promoter analyses indicate substantial functional diversification within the *LdERF* family and provide the basis for investigating the functions of individual gene members in specific developmental processes, including bulblet regeneration.

### 3.2. The LdERF Gene Family Is Widely Involved in Bulblet Regeneration in Lanzhou Lily

Plant regeneration is a dynamic developmental process during which differentiated somatic cells reacquire developmental competence, undergo cell proliferation, and subsequently establish new meristematic structures that give rise to regenerated organs. This process is coordinately regulated by TFs and multiple phytohormone signaling pathways. Increasing evidence indicates that ERF family members participate in plant regeneration by regulating cell division, hormonal responses, and the expression of regeneration-associated genes. However, regenerative capacity and the underlying molecular mechanisms vary substantially among plant species and developmental systems.

Thus far, the molecular regulatory networks governing plant regeneration have been most thoroughly elucidated in dicotyledonous species, especially *A. thaliana* and cotton [27,28]. In these systems, auxin signaling, WOX family members, and WUS-mediated establishment and maintenance of meristematic tissues constitute important regulatory modules controlling regeneration [29,30]. In monocotyledonous plants, including rice and maize, regeneration is likewise regulated by the coordinated activities of morphogenic TFs, including AP2/ERF and WOX family members, together with phytohormone signaling pathways [27,31,32,33]. These findings suggest that although the specific regulatory components may differ among species, partially conserved transcriptional and hormonal networks may also contribute to bulblet regeneration in monocotyledonous bulbous plants such as lily [34].

Transcriptome profiling in this study demonstrated dynamic expression patterns of numerous *LdERF* genes during bulblet regeneration. Different family members displayed distinct expression profiles, with some genes showing high expression during the early stages of regeneration followed by a gradual decline, whereas others progressively accumulated transcripts during later stages. Such stage-specific expression patterns suggest that different *LdERF* members may function at different phases of bulblet regeneration, potentially contributing to the regulation of regenerative competence, cell proliferation, meristem establishment, and bulblet development.

Among the identified genes, *LdERF179* showed relatively high expression in the initial phases of bulblet regeneration, followed by a gradual decline as regeneration progressed. This result was consistent with *AtERF115* and *AtWIND1* being rapidly induced in response to tissue injury and promoting cellular reprogramming, callus formation, stem cell activation, and organ regeneration [35,36]. The early accumulation of *LdERF179* transcripts therefore suggests that this gene may participate in the initiation of bulblet regeneration, potentially by promoting the acquisition of regenerative competence in lily scale cells.

In addition to their dynamic expression during regeneration, several *LdERF* genes exhibited pronounced tissue-specific expression patterns. These observations suggest that the *LdERF* family may participate not only in bulblet regeneration but also in the development and physiological regulation of roots, leaves, scales, and floral organs. Collectively, these results underscore the functional versatility of the *LdERF* family and identify *LdERF179* as a candidate regulator of the early stages of bulblet regeneration.

### 3.3. LdERF179 Promoted Bulblet Regeneration in Lanzhou Lily

Functional analyses further demonstrated that *LdERF179* plays a positive role in bulblet regeneration. Silencing of *LdERF179* using VIGS resulted in a pronounced reduction in bulblet regeneration frequency, which decreased from 40.67% in the control to 9.43% following *LdERF179* silencing. Conversely, overexpression of *LdERF179* enhanced the expression of several genes associated with auxin signaling and regeneration. Together, these data suggested that *LdERF179* may influence the regeneration of bulblets in Lanzhou lily by regulating auxin signaling and the expression of key regeneration genes.

Histological observations provided further evidence for this function. The basal regions of control scales developed compact meristematic protrusions consisting of actively dividing cells, whereas *LdERF179*-silenced scales exhibited loosely arranged and enlarged cells with only limited protrusion formation. These cellular and structural differences suggest that reduced *LdERF179* expression compromises the cellular reprogramming and meristematic activity required for bulblet initiation. In addition, *LdERF179* was localized in the nucleus, further supporting its function as a transcriptional regulator.

The involvement of ERF proteins in regeneration has also been documented in other plant species. For example, *ERF5* promotes protocorm-like body regeneration in *Dendrobium officinale* by enhancing the expression of *STM* [37], whereas *OsERF71* positively regulates organ regeneration in rice [38]. Consistent with these findings, our results indicate that *LdERF179* also promotes organ regeneration, suggesting that the regeneration-related functions of ERF proteins may be partially conserved across diverse plant species.

Phytohormones, particularly auxin, play central roles in plant organ regeneration. Local auxin accumulation can promote cellular dedifferentiation, cell proliferation, and the establishment of new meristematic tissues during regeneration [39]. In the present study, *LdERF179* silencing markedly decreased the expression of multiple genes linked to auxin biosynthesis, transport, and signaling, such as *LdASA1*, *LdAUX1*, and *LdIAA27*. *ASA1* participates in tryptophan biosynthesis, supplying a key precursor for auxin synthesis [40], whereas *AUX1* functions as an auxin influx carrier involved in cellular auxin transport [41]. In addition, *IAA27*, an Aux/IAA family member, participates in auxin signaling through interactions with ARF TFs [42]. The simultaneous suppression of these genes upon *LdERF179* silencing indicates that *LdERF179* may influence the auxin regulatory network directly or indirectly during bulblet regeneration.

*LdERF179* silencing also markedly decreased the expression of *LdWOX11* and *LdPIN1*, further connecting this TF to known regeneration-related regulatory pathways. *WOX11* is crucial in the initial phases of organ regeneration and the acquisition of regenerative cell fate [43], while auxin transport mediated by PIN facilitates the formation of local auxin gradients necessary for organ initiation. Thus, the simultaneous reduction in *LdAUX1*, *LdPIN1*, *LdIAA27*, *LdASA1*, and *LdWOX11* expression suggests that *LdERF179* may contribute to bulblet regeneration by coordinating auxin-related processes with the activation of regeneration-associated developmental programs.

Based on these findings, *LdERF179* may function upstream of, or in coordination with, auxin-responsive and WOX-associated regulatory networks during bulblet initiation. However, the present results are based primarily on expression changes following gene silencing and overexpression and therefore do not establish direct regulatory relationships. Further investigations, including promoter-binding and transcriptional activation assays, are necessary to confirm if these genes serve as direct transcriptional targets of *LdERF179*.

Promoter analysis revealed the presence of putative ERF-associated *cis*-regulatory elements in several auxin- and regeneration-related genes. Together with the coordinated changes in their expression following manipulation of *LdERF179*, these observations raise the possibility that *LdERF179* may directly or indirectly regulate their transcription. Thus, a working model is proposed wherein *LdERF179* facilitates bulblet regeneration by regulating auxin biosynthesis, transport, and signaling, as well as modulating the expression of key regeneration-related genes. Further identification of the direct downstream targets of *LdERF179* will be essential for defining the precise molecular mechanism through which this TF regulates bulblet regeneration in Lanzhou lily.

## 4. Materials and Methods

### 4.1. Plant Materials

Healthy bulblets of Lanzhou lily at five developmental stages (S0–S4), free of pests and diseases, were obtained from the Biotechnology research institute, Fujian Academy of Agricultural Sciences, and stored at 4 °C prior to use. For bulblet regeneration, bulbs with a circumference of 14–16 cm were selected, and the outer three to four layers of scales were removed. Middle-layer scales were excised and embedded in a substrate (Peat soil:vermiculite (*v*/*v*) = 3:1), moisture conditions were characterized by a moist substrate (~30% humidity), and scales were cultured in darkness at 24 °C. The scale induction (SI) stage corresponds to freshly detached scales (0 d, S0). The regeneration initiation (RI) stage (7 d, S1) was characterized by the appearance of slight morphological changes at the basal region of the scales. The promeristem formation (PF) stage (18 d, S2) indicates the emergence of visible protrusions at the scale base. The leaf primordium (LP) stage (22 d, S3) corresponded to the enlargement of these protrusions into small bulblets, whereas the bulblet formation (BF) stage (26 d, S4) was identified by the formation of distinct bulblet structures with recognizable bulb scales. For sample collection, from the basal region of each scale, approximately 0.5 cm of tissue was excised, immediately immersed in liquid nitrogen, and stored for RNA extraction. Three biological replicates were carried out for each stage, with 30 scales for each replicate. The gathered samples were later employed to examine the gene expression profiles during various phases of bulblet regeneration.

### 4.2. LdERF Gene Family Identification in Lanzhou Lily

The genome sequence of *Lilium davidii* var. *unicolor* was obtained via the China National Sequence Archive (CNSA; assembly accession CNA0139751) from the China National GeneBank DataBase (CNGBdb). From The Arabidopsis Information Resource (TAIR) database, the protein sequences of four representative AP2/ERF family members from *Arabidopsis thaliana* (AT1G06160, AT5G61590, AT2G46310, and AT2G20880) were retrieved. To find members of the *LdERF* gene family, the hidden Markov model (HMM) profile of the AP2 domain (PF00847) was obtained from the Pfam database and served as the query for a genome-wide search via the Simple HMM Search tool in TBtools v2.487, with an E-value cutoff of 1 × 10^−10^ [44]. Simultaneously, TBtools was used for BLASTP analysis of *A. thaliana* AP2/ERF protein sequences and predicted Lanzhou lily protein sequences, setting E-value cutoff at 1 × 10^−10^ [44]. The candidate genes identified by the HMM search and BLASTP analysis were intersected, and redundant sequences were removed to obtain the preliminary set of *LdERF* candidate genes. Next, the SMART, NCBI Conserved Domain Database (CDD), confirmed the conserved AP2 domains of the candidate proteins and Pfam databases. Sequences lacking a complete AP2 domain, containing truncated protein sequences, or lacking an entire open reading frame (ORF) were excluded. The remaining genes were finally identified as members of the *LdERF* subfamily.

### 4.3. Chromosomal Localization, Synteny Analysis, Gene Structure Analysis and Conserved Motif Identification of the LdERF Subfamily

*LdERF* genes’ chromosomal locations were derived from the genome annotation file. Chromosomal distribution maps were generated using the Gene Location Visualize function in TBtools [44], and the *LdERF* genes were named based on their physical positions on the chromosomes. Gene duplication and synteny analyses were conducted with the MCScanX Super Fast module integrated in TBtools with the default parameters: CPU for BlastP = 2, E value = 1 × 10^−10^, Num of BlastHit = 5, and syntenic relationships between *Lilium davidii* var. *unicolor*, *Arabidopsis thaliana*, and rice (*Oryza sativa*) were visualized.

Gene structure analysis was conducted by extracting the coding sequences (CDSs) and genome annotation file corresponding to genomic sequences, and exon–intron organization was visualized using TBtools [44]. Conserved motifs of the LdERF proteins, with the maximum number of motifs set to 10, were identified using the MEME Suite, while every other parameter was maintained at their default settings. The identified motifs were subsequently visualized using TBtools.

### 4.4. Cis-Regulatory Element and Phylogenetic Analyses of the LdERF Subfamily

From the genome annotation file, the 2000 bp genomic sequences upstream of the translation initiation codon (ATG) of each *LdERF* gene were extracted and submitted to the PlantCARE database for predicting *cis*-regulatory elements. The identified cis-elements were visualized using TBtools, and the numbers and distributions of light-responsive, stress-responsive, and phytohormone-responsive *cis*-elements were statistically analyzed [44].

To explore the evolutionary connections of the LdERF proteins, the amino acid sequences of the identified LdERF family members and the 65 ERF proteins from Arabidopsis thaliana were aligned and imported into MEGA version 11.0 for phylogenetic analysis. A phylogenetic tree was built via the Maximum Likelihood (ML) approach with 1000 bootstrap replicates, the best-fit substitution model was determined based on the Bayesian Information Criterion (BIC), and the Jones–Taylor–Thornton (JTT) model with Gamma-distributed rates (+G) was selected as the optimal model. The resulting tree was then annotated and visualized via the Interactive Tree of Life (iTOL) online platform.

### 4.5. LdERF Genes Expression Profiling in Different Tissues and During Bulblet Regeneration, and Validation by RT-qPCR

Transcriptome datasets generated from five developmental stages of bulblet regeneration (S0–S4) (PRJCA060256) and six different tissues (root, stem, leaf, flower, bud, and scale) of *Lilium davidii* var. *unicolor* (PRJCA068453) were utilized to examine the expression profiles of *LdERF* genes. Three independent biological samples were included in the transcriptomic data for each stage. The FPKM (fragments per kilobase of transcript per million mapped reads) values were normalized using the log_2_ (FPKM + 1) transformation. Genes with an FPKM value of zero in all samples were excluded, and the normalized expression data were visualized as heatmaps using TBtools. Genes exhibiting comparatively elevated expression in the initial phases of bulblet regeneration were selected as candidate genes for subsequent quantitative real-time PCR (RT-qPCR) validation.

Using the FastPure Plant Total RNA Isolation Kit (Polysaccharides & Polyphenolics-rich) (Vazyme, Nanjing, China), total RNA was isolated from the basal parts of lily scales gathered at five stages of bulblet regeneration, as per the maker’s guidelines. The HiScript III All-in-one RT SuperMix Perfect for qPCR kit (Vazyme, Nanjing, China) was utilized to synthesize first-strand cDNA, which served as the template for RT-qPCR analysis.

To ascertain the expression levels of nine *LdERF* genes at stages S0–S4, RT-qPCR was performed using Taq Pro Universal SYBR qPCR Master Mix (Vazyme, Nanjing, China). Each 10 µL reaction contained 5 µL of Taq Pro Universal SYBR qPCR Master Mix, 0.2 µL of each gene-specific primer (10 µM), 0.5 µL of diluted cDNA template, and 4.1 µL of nuclease-free water. The cycling conditions consisted of an initial denaturation at 95 °C for 3 min, followed by 40 cycles of 95 °C for 10 s, 60 °C for 30 s, and 72 °C for 15 s. A melt-curve analysis was then performed with 60 °C for 1 min, followed by 95 °C for 15 s. Melt curves for all primer pairs showed a single, sharp peak, confirming the specificity of amplification and the absence of primer dimers or non-specific products. Primer amplification efficiencies were determined from standard curves generated with a 10-fold serial dilution of pooled cDNA, and the efficiencies of all primer pairs ranged from 90 to 100%. LdActin served as the internal reference gene. The reactions were performed using a QuantStudio™ 1 Plus Real-Time PCR System (Thermo Fisher Scientific, Waltham, MA, USA). The 2^−ΔΔCt^ method was used to calculate relative gene expression levels. Each experiment included three biological replicates, with three technical replicates for each biological sample. GraphPad Prism version 10.5.0 was utilized for statistical analyses and graphical visualization. The GenScript Real-time PCR Primer Design Tool was employed to design primers in this study, listed in Table S6.

### 4.6. Subcellular Localization Analysis

The WoLF PSORT website (https://wolfpsort.hgc.jp/, accessed on 1 August 2026) was used to perform subcellular localization prediction. BamH I and Sal I were chosen as double restriction enzyme sites to construct the recombinant 35S::*LdERF179*-eGFP fusion vector. The correctly assembled construct was confirmed by sequencing analysis performed by Shangya Biotechnology Co., Ltd.

Using the freeze–thaw method, the recombinant plasmid and empty vector were introduced into *Agrobacterium tumefaciens* strain GV3101. Single colonies were selected and cultured for activation, and the OD_600_ of the bacterial suspension was adjusted to 0.6–0.8. The cultures were kept at room temperature for 2 h before being used for transient transformation.

Fresh onion bulbs of similar size were selected, and the inner epidermis of the onion bulb scales was transiently transformed by injection infiltration. Following infiltration, the samples were kept in darkness for 48–72 h. The injected inner epidermal tissues were then collected and stained with DAPI staining solution (Horie Biotechnology, Shanghai, China) for 5–10 min, then rinsed 2–3 times using sterile water to remove residual dye.

Temporary slides were prepared, and the eGFP fluorescence signal distribution in the onion inner epidermal cells was observed under a 20× objective in 488 nm using a laser confocal scanning microscope (Leica, Wetzlar, Germany). Images were captured with the following settings: Format 1024 × 1024, Speed 400, and exported in TIFF format. Based on the fluorescence co-localization results, the subcellular localization of the *LdERF179*-encoded protein was determined in plant cells.

### 4.7. Virus-Induced Gene Silencing (VIGS) of LdERF179 and Phenotypic Analysis

A fragment (261 bp, to be specific) of *LdERF179* was amplified from the genomic sequence with primers TRV2-*LdERF179*-CF and TRV2-*LdERF179*-CR; the fragment sequence is shown in Table S7. The amplified fragment was placed into the pTRV2 vector utilizing Sal I and BamH I restriction sites to generate the recombinant vector pTRV2-*LdERF179*, which was subsequently transformed into *Agrobacterium tumefaciens* strain GV3101 (Weidi Biotechnology Co., Shanghai, China). The recombinant construct was confirmed by DNA sequencing.

Uniform middle scales of Lanzhou lily were selected as experimental materials. Two treatments were established: the empty-vector control (TRV1 + TRV2) and the gene-silencing treatment (TRV1 + TRV2-*LdERF179*).

Carrying TRV1, TRV2, or TRV2-*LdERF179* Agrobacterium cultures were grown overnight in LB medium combined with the appropriate antibiotics at 28 °C with shaking at 180 rpm. By centrifugation at 7000 rpm for 10 min, bacterial cells were collected, resuspended in infiltration buffer containing 10 mM MgCl_2_, 10 mM MES (pH 5.8), and 200 μM acetosyringone (AS), and adjusted to an OD_600_ of 0.6–0.8. Before infiltration the suspensions were incubated in the dark for 2 h.

Equal volumes of TRV1 and TRV2 (or TRV2-*LdERF179*) cultures were mixed (1:1, *v*/*v*). Lily scales were completely immersed in the infiltration suspension and vacuum infiltrated at 0.8 KPa for 15 min, with the pressure released slowly for 15 min. This infiltration cycle was repeated once. After infiltration, the scales were air-dried and cultured on moist absorbent cotton under appropriate growth conditions, dark conditions at 25 °C. Each treatment was performed with 3 biological replicates, and each replicate infected 30 scales.

After 21 days, phenotypic statistics were performed and morphological changes were observed and photographed under a stereomicroscope. Total RNA was isolated from both control and silenced samples, and RT-qPCR was conducted to assess the silencing efficiency of *LdERF179* and the expression levels of auxin metabolism- and regeneration-related genes.

### 4.8. Overexpression of LdERF179 in Tissue-Cultured Bulblets of Lanzhou Lily

Utilizing BamHI and SalI sites, the *LdERF179* full-length coding sequence was cloned into pCAMBIA1301 binary vector driven by CaMV 35S promoter, generating the 35S::*LdERF179* overexpression construct. The pCAMBIA1301 vector was given by the Biotechnology research institute, Fujian Academy of Agricultural Sciences. The recombinant plasmid was verified by PCR after being introduced into *Agrobacterium tumefaciens* strain EHA105 via the freeze–thaw method, then stored at −80 °C.

Genetic transformation was performed according to the highly efficient transformation system previously established for Oriental lily [45]. Tissue-cultured plantlets of the Oriental lily cultivar ‘Siberia’ were used as transformation materials. Bulb scales were cut into ultrathin sections (0.5–1.0 mm thick) and pre-cultured on MS medium supplemented with 0.1 mg L^−1^ 6-benzyladenine (6-BA) and 0.1 mg L^−1^ α-naphthaleneacetic acid (NAA) for 3 days. Prior to Agrobacterium infection, the explants were air-dried for 30 min.

The explants were soaked in the Agrobacterium suspension and endured vacuum infiltration at 0.8 KPa for 15 min, then the pressure was released slowly for 15 min. The infiltration procedure was repeated once. After infiltration, the explants were air-dried and transferred to co-cultivation medium consisting of MS supplemented with 0.1 mg L^−1^ 6-BA, 0.1 mg L^−1^ NAA, and 200 μM acetosyringone, followed by incubation for 3 days. Three biological replicates were set per group, with each replicate infecting 30 ultrathin sections inoculated per Petri dish.

Total RNA extraction method, RT-qPCR method, and statistical analyses and graphical visualization are referenced in Section 4.5 of this study.

### 4.9. Paraffin Sectioning and Microscopic Observation

The basal regions of bulblets from *LdERF179*-silenced plants were excised and cut into approximately 2 mm × 2 mm tissue blocks. The samples were immediately fixed in FAA fixative at room temperature for subsequent paraffin embedding, sectioning, and microscopic observation.

### 4.10. Statistical Analysis

GraphPad Prism version 10.5.0 was used to perform all statistical analyses. Data were represented as the mean ± SE for three replicates (*n* = 3). Differences among treatments were evaluated by one-way analysis of variance (ANOVA); asterisks from Student’s *t*-test: * *p* < 0.05, ** *p* < 0.01. Lowercase letters indicate significant differences at *p* < 0.05.

## 5. Conclusions

In this study, 206 *LdERF* genes were systematically identified from the *Lilium davidii* var. *unicolor* genome. Comprehensive analyses, including phylogenetic relationships, gene structure, conserved motifs, chromosomal distribution, synteny, and *cis*-regulatory elements, revealed the evolutionary characteristics and structural diversity of the *LdERF* gene family. Expression profiling demonstrated that *LdERF* genes are widely involved in bulblet regeneration and tissue development, and *LdERF179* exhibited relatively high expression during the early stage of bulblet induction.

Functional analyses further demonstrated that silencing of *LdERF179* markedly inhibited bulblet regeneration, whereas overexpression of *LdERF179* upregulated auxin-related genes and regeneration-related genes, such as *LdASA1*, *LdAUX1*, *LdIAA27*, *LdWOX11*, and *LdPIN1*. This indicated that *LdERF179* may promote bulblet regeneration by coordinating auxin signaling with regeneration-related regulatory pathways. The specific mechanism by which *LdERF179* regulates auxin metabolism requires further in-depth functional validation.

Overall, this study offers a thorough characterization of the *LdERF* gene family in Lanzhou lily and *LdERF179* is considered a promising candidate gene for improving regeneration efficiency and for applications in molecular breeding. *LdERF179* may promote bulblet regeneration by coordinating auxin signaling with regeneration-related regulatory pathways, and subsequent research can further investigate how *LdERF179* regulates the auxin metabolic pathway to control bulb regeneration. These findings offer valuable insights into the molecular mechanisms underlying bulblet regeneration and offer a promising candidate gene for improving regeneration efficiency and developing genetic transformation and molecular breeding strategies in lily.

## Data Availability

The RNA-seq datasets generated and analyzed in this study have been deposited in the National Genomics Data Center (NGDC) under accession numbers PRJCA060256 and PRJCA068453.

## Supplementary Materials

The following supporting information can be downloaded at: https://www.mdpi.com/article/10.3390/plants15213265/s1, Figure S1. The distribution of *LdERF* genes on chromosomes; Figure S2. Gene structure of *LdERF* family genes; Figure S3. Conserved motif composition and domain organization of LdERF proteins in *Lilium davidii* var *unicolor*; Figure S4. Analysis of *cis*-regulatory elements in the promoter regions of LdERF genes in *Lilium davidii* var *unicolor*; Figure S5. PCR detection of the TRV2 coat protein (CP) gene in VIGS plants; Figure S6. PCR identification of the hygromycin resistance gene (HYG) in 1301-LdERF179 transgenic lines; Table S1. Physicochemical properties of LdERF proteins; Table S2. Orthologous relationships between LdERF genes and Arabidopsis/Rice ERF genes; Table S3. Statistics of *cis*-acting elements in the LdERF family of *Lilium davidii* var. *unicolor*; Table S4. Count and FPKM values of LdERF179 across three biological replicates in the transcriptome datasets of different tissues and bulblet regeneration stages of Lilium ‘Siberia’; Table S5. The function of LdERF179 in the scale-induced bulblet regeneration verified by virus-mediated gene silencing technology; Table S6. Primers in this study; Table S7. LdERF179 VIGS fragment sequence and insert length.
